# An Educational Game to Teach Children about Air Quality Using Augmented Reality and Tangible Interaction with Sensors

**DOI:** 10.3390/ijerph20053814

**Published:** 2023-02-21

**Authors:** João Fernandes, Tomás Brandão, Susana Marta Almeida, Pedro Santana

**Affiliations:** 1 ISCTE, Instituto Universitário de Lisboa (ISCTE-IUL), Av. das Forças Armadas, 1649-026 Lisboa, Portugal; 2 ISTAR—Information Sciences and Technologies and Architecture Research Center, Av. das Forças Armadas, 1649-026 Lisboa, Portugal; 3 Centro de Ciências e Tecnologias Nucleares, Instituto Superior Técnico, Universidade de Lisboa, Estrada Nacional 10, 2695-066 Bobadela, Portugal

**Keywords:** air quality, augmented reality, child–computer interaction, educational games, serious games, tangible interaction

## Abstract

Air pollution is known to be one of the main causes of injuries to the respiratory system and even premature death. Gases, particles, and biological compounds affect not only the air we breathe outdoors, but also indoors. Children are highly affected by the poor quality of the air they breathe because their organs and immune systems are still in the developmental stages. To contribute to raising children’s awareness to these concerns, this article presents the design, implementation, and experimental validation of an serious augmented reality game for children to playfully learn about air quality by interacting with physical sensor nodes. The game presents visual representations of the pollutants measured by the sensor node, rendering tangible the invisible. Causal knowledge is elicited by stimulating the children to expose real-life objects (e.g., candles) to the sensor node. The playful experience is amplified by letting children play in pairs. The game was evaluated using the Wizard of Oz method in a sample of 27 children aged between 7 and 11 years. The results show that the proposed game, in addition to improving children’s knowledge about indoor air pollution, is also perceived by them as easy to use and a useful learning tool that they would like to continue using, even in other educational contexts.

## 1. Introduction

The World Health Organization (WHO) defines air pollution as the “contamination of the indoor or outdoor environment by any chemical, physical or biological agent that modifies the natural characteristics of the atmosphere” [1]. These compounds affect the air we breathe and are associated with millions of premature deaths and the cases of diseases such as cancer and obstructive pulmonary disease every year [1], as well as increasing infant mortality rates [2]. To achieve both economic development and emission reduction, prioritizing ecological conservation and boosting green development is key [3].

Indoor air represents a considerable part of daily exposure to pollution, since daily life is nowadays mostly spent indoors. In indoor environments, the concentrations of polluting compounds can actually be higher than those outside due to the closer proximity to emitting sources and lower pollutants’ dilution. Children are especially vulnerable to air pollution, as their respiratory rate is higher than in adulthood and their immune system is still developing. When exposed to air pollution for long periods of time, the risk of developing or aggravating respiratory pathologies increases considerably [4]. Hence, it is pivotal to protect children from this often invisible and difficult to identify peril.

If we can make children more aware of air pollution and its health impacts, we may be able to promote healthier and environmentally protective behaviors for these children. In fact, the influence of air pollution on public health diminishes as the education level increases [2]. Raising children’s awareness is particularly advantageous in the first years of school activity, as investing in learning at a younger age is known to bring positive effects in later life [5]. Awareness can be raised with traditional learning methods, such as formal classes, books, and documentaries. On the other hand, new interactive digital technologies offer new opportunities for active and customized learning activities towards raising the motivation for learning. It is important to continually innovate in the way teaching methods and tools are created and that they include differentiating factors that induce a surprise factor and help make the experience memorable, especially for primary school children [6]. Investing in quality education during childhood makes children less likely to fail grades and reduces the chances of inclusive and special education needs. Moreover, children are more likely to successfully complete secondary education and be part of the professional market with above-average wages [5].

Educational games are an example of how interactive digital technologies can be used to create immersive experiments that exploit children’s intrinsic motivations (e.g., attraction for novelty, play, and stories) to raise their willingness to learn concepts that could be otherwise unpleasant to learn. Using virtual reality (VR), educational games can be designed to immerse the player in a simulated world, amplifying one’s sense of presence in that world. However, virtually sending our body to another place can be uncomfortable for some [7]. Moreover, full immersion in a virtual world hampers the ability of students to socially interact in the physical world with their colleagues throughout the experiment. Augmented reality (AR) is a promising add-on to educational games, as it allows students to experience virtual content in the real world that would otherwise be invisible (e.g., air constituents) and to interact with virtual content using physical objects (i.e., tangible interfaces), while physically and socially interacting with their colleagues.

In this article, we present an AR-based educational game that aims to provide an interactive learning experience about indoor air quality for elementary school children, to be played in pairs, in the classroom. The goal is to teach children how everyday objects contribute to households’ air quality. To meet this goal, children are asked to present physical objects to a physical sensor node capable of monitoring the air quality. In return, children are able to see the physical sensor node augmented with virtual representations of the object’s emanated air pollutants measured by the sensor node, rendering visible the invisible. The game was implemented in Unity/Vuforia and interacts with a sensor node developed in the ExpoLIS project [8].

The game was evaluated with a Wizard of Oz experiment with a sample of 27 children aged between 7 and 11 years. Herein, the air quality measurements were covertly generated in real-time by a human being instead of measured by the sensor node. This option reduces the variance in user testing resulting from the natural stochastic nature of both air diffusion and sensing. The experiments were designed to test four research hypotheses: H1—AR games are able to teach children about the causes of indoor air pollution; H2—AR games are able to teach children about the mechanisms available to clear indoor air pollution; H3—AR games provide a satisfying and emotionally stimulating air pollution learning experience, with high replay value; and H4—AR games benefit from allowing the child to interact with real pollution sources instead of card-based representations. The obtained results confirm the four research hypotheses, showing that the proposed AR-based learning game, in addition to improving children’s knowledge about indoor air pollution, is also perceived by them as easy to use and a useful learning tool that they would like to continue using, even in other educational contexts.

This article is organized as follows. Section 2 presents a literature survey of related work. Then, in Section 3, the developed AR-based game is described alongside its key implementation details. Section 4 presents the experimental setup and results. Finally, Section 5 draws conclusions and provides future work directions.

## 2. Related Work

### 2.1. Educational Games in Learning

The use of games in the educational context is an active research topic with especially positive results in younger age groups [9]. Educational games, a sub-genre of serious games, show promise in knowledge transmission as well as students’ commitment, motivation, and capacity to retain the acquired knowledge [10]. Combining teaching strategies with game design may guide learners through complex tasks and new concepts in a relaxed and pleasant way. Adequate game-based learning should enable autonomous learning at the user’s own pace and boost one’s self-motivation [11].

When designing a game, one must need to choose between single-player and multi-player and, for the latter, between cooperative and competitive strategies. Previous studies shed some light about which options are the most likely to deliver the best results when teaching is the ultimate goal of the game. In the study presented in [12], students showed higher rates of participation in classes after playing cooperative games, when compared to after playing competitive games [12]. These data are in line with the study presented in [11], which revealed that students better enjoy games when these are played in groups, cooperatively. Furthermore, [13] investigated the effect of using collaborative methods for learning mathematics in children with and without learning difficulties. With a collaborative group working in pairs and a competitive group working individually, it was observed that the collaborative group achieved the best outcomes. In addition, the peers in the collaborative group established positive relationships with each other, helping and encouraging each other.

Nevertheless, competition encourages personal development and improvement. However, the inseparable need for a winner and a loser leads users to focus not only on their individual success, but also on their opponent’s failure. As such, the cooperative vision assumes a more enriching role, since it does not promote tense environments or aggression between the participants. It also reinforces the ability of users to relate positively to each other, creating a suitable empathy and trust environment, and encourages the development of communication skills, which are extremely important for success in today’s society. In addition, it helps develop interpersonal relationships and is even associated with more successful professional careers [14].

### 2.2. Augmented Reality in Educational Games

Several studies have demonstrated the ability of computing technologies to persuade and influence their users’ behaviors [11]. AR games contribute to this capacity, being especially useful for teaching science, to represent abstract and difficult to visualize subjects [15], and to develop computational thinking skills [16]. Developing computational thinking is important for children to be able to reason about the global consequences of their local actions. AR games have the potential to integrate abstract and difficult-to-interpret information from the real world. This eases the creation of theory–practice links, allowing interaction in real contexts and learning through execution. Problems of traditional education, such as the student’s lack of focus and distractions, are important reasons that lead teachers and educators to find new ways for knowledge transmission that adapt to children’s needs [17]. There have been many demonstrations that AR is able to compensate for some gaps of traditional teaching [17,18]. AR potentiates a more informal learning environment, allowing learners to interact with the technology as if they were playing, which benefits knowledge acquisition [11]. Learners also feel that AR-based learning is a more efficient and motivating way of acquiring knowledge than traditional teaching methods [19]. Furthermore, AR can assist the learning process for children with disabilities, such as autism, helping them stay focused [20], and to attract them for behavioral therapies [21].

In the educational context, AR is currently most often developed for mobile devices [22], largely due to ease of use and accessibility, as they have a tactile interface. Additionally, mobile devices are affordable as well as easy to acquire and to replace. However, the small size of the screen constrains the amount of information that can be presented. Another disadvantage of mobile devices is the frequency of distractions, especially when used for didactic purposes on child audiences [19,23]. Head-mounted displays (HMD) are an alternative to mobile devices, but their considerable weight and cost are limiting factors for the purpose of educating children. Moreover, HMD may hamper the feeling of co-presence in collaborative scenarios. Therefore, the use of desktop computers equipped with webcams emerges as an interesting alternative to mobile- and HMD-based AR in educational contexts.

Cognitive overload resulting from an excessive presentation of information to the learner, is a well-known key challenge when developing AR-based educational tools [24], contributing to the reduction in student’s learning efficiency, focus and motivation. However, when properly designed and validated, AR can be an extremely useful tool to overcome both the information overload and the lack of motivation associated to traditional teaching methods [25,26], serving as creative unlockers [27], improving visualization skills [26], and helping understand difficult to relate concepts [19]. When developing AR-based tools for students, it is also important to bear in mind that the cognitive ability, individual learning style, spatial visualization ability, and previous experiences with these types of learning environments are factors that influence the effectiveness of the teaching techniques [25].

Ideally, the designers of educational interactive tools should be provided with a set of design guidelines, leading the devised tool to meet the adequate teaching effectiveness. In [15], a set of design principles for AR-based learning tools is presented, based on an analysis of existing literature. The first principle, “captivate and then challenge”, aims to prevent users from suffering from information overload or feeling that they are not able to deal with the challenge. It is essential to start by outlining strategies to guide users through the most elementary concepts and mechanics of the experience and, only then, to challenge them with more complex problems, ideally adapted to the game’s progress phase and the user’s performance. The second principle, “guide the experience through the game’s story”, aims to guide the user’s learning process and one’s interactions with the system through immersive narratives strategically designed for this purpose, building a bridge between entertainment and the ability to transmit educational content. The narrative should include characters that appear at key moments to provide context, tasks, and guide the user’s attention. Scoring systems that reward or penalize their actions, directing you to the idealized outcome, are also important elements to include. The third principle, “see the invisible”, is directly related to the basic functionality that AR provides, that is, augmenting the real world with visual representations of content that would otherwise be invisible to the observer. In a metaphorical sense, the AR tool operates as a lenses.

### 2.3. Augmented Reality Meets Sensors

Sensor networks and Internet of Things (IoT) are widely applied in the monitoring of real phenomena, such as air quality [8]. The data generated by these devices contain information relevant to raise children’s and young people’s awareness of major societal issues, such as environmental monitoring. Providing young people with access to air quality data through immersive 3D environments exhibiting a video game appearance is advantageous when compared to traditional visualization techniques [28]. The use of game engines as explorative, low-entry tools for visualizing complex air pollution datasets is further discussed in [29].

Augmented reality is also recognized as an advantageous means of visualizing, controlling, and interacting with IoT devices [30]. For instance, the awareness of energy consumption among students can be improved using mobile AR to present sensor data gathered from buildings [6]. AR and IoT can also be orchestrated for helping people with reduced mobility to become more independent in performing daily activities, such as choosing products from a supermarket shelf [31]. To enable the observation of electromagnetic radiation emitted by ordinary electronic equipment, present in a room where users can move around and freely explore the environment, an AR experience using an HMD was developed [32]. The results collected by the authors show the validation and appreciation of the participants regarding the use of these techniques for the visualization and learning of invisible content. AR can also be used to teach children about colors by enabling them to point a color sensor at any object in the real world and obtain its color in return [33].

The potential of mobile AR to create attractive and interesting gaming experiences for the presentation of air quality data was demonstrated in [34]. The proposed game consisted of rewarding the user whenever the temperature and CO_2_ levels from a distant location (where an IoT device is installed) are properly guessed. Clues are provided to the user by the dressing of a virtual character (t-shirt vs. scarf, with or without a gas mask). Users found the experience interesting and fun, promoting awareness of environmental problems. More recently, a study where the residents of a building monitor the air quality of their homes based on the data acquired by an IoT network using AR was carried out [35]. When compared with non-AR users, AR users showed a higher degree of satisfaction regarding quality of experience and effectiveness of the presented information.

### 2.4. User Input in Augmented Reality

In most AR applications, users need to provide some form of input. For instance, the MagicHand project [36] allows users to control IoT equipment via hand gestures. Alternatively, the user may interact with the system by manipulating real objects, i.e., using tangible interfaces. Tangible interfaces take advantage of the user’s natural skills for manipulating physical objects, providing a greater degree of immersion by including sensory stimulation mechanisms, such as haptic, weight, texture, and temperature sensations [37,38]. In fact, a comparative study between touchscreens, tangible interfaces, and classic mouse–keyboard interaction concluded that the user preference and interaction speed showed the best results for tangible interfaces [39]. Moreover, in an AR-based educational experiment where children were challenged to relate real plants to their fruits and leaves, the use of real objects was considered to be a factor that provides a strong contribution to the learning experience [40].

Although object detection, segmentation, and tracking is becoming ubiquitous, these remains challenging tasks when objects are being manipulated by users in the wild. To mitigate some of these problems, particularly those related to occlusions, multi-camera settings are often employed [41,42]. Some of the manipulated physical objects can be used as pointers for the user to direct the system’s attention. A pointer can be as simple as a stick with a colored sphere for simple vision-based tracking [42]. For a more robust tracking and pose estimation, the tip of the stick can be attached to a visual marker [43]. Visual markers can be used to track the pose of other types of objects, such as books, and even to operate as buttons (pressed by the occlusion of the marker) or sliders (sliding by moving the marker) [44].

Although the appearance of visual markers is often inconsistent with the game’s overall aesthetics, which may impact the user’s sense of presence if not properly hidden by the virtual augmentations, their detection is well understood, affordable, and simple to implement. Therefore, the marker-based tracking of physical objects still represents the most accessible method for prototyping tangible interfaces and, thus, particularly suited for low-budget classroom contexts.

### 2.5. Discussion

The presented literature survey highlights the value of AR in the design of educational games and of user interfaces for sensor networks and IoT devices. The survey also reveals the advantages of considering physical objects as tangible interfaces. Our educational game, presented herein, combines and extends these findings. In particular, the game provides children with the possibility of interacting with air quality-sensing devices via the manipulation of everyday physical objects. This novel interaction possibility, not addressed by previous work, is intended to render tangible the invisible and, thus, to increase the learning gain. By allying it with a playful gaming experience, the emotional involvement in the learning experience is expected to grow and, hence increasing the chances that the child is willing to fully complete it and even to repeat it. To facilitate accessibility and cooperative gameplay in the classroom context, the game follows the desktop-based AR paradigm and relies on visual markers. The game was designed to be a cooperative multi-player, building upon the identified advantages of collaboration over competition in educational contexts.

## 3. Gamified Experience Description

### 3.1. Overview

The gamified experience described in this section combines the use of augmented reality with air quality measurements, creating an instrument that allows a child to visualize and interact with elements taken from both the virtual and real worlds. Similarly to microscopes or X-ray machines that allow observations at different scales/spaces, the idea is to create a gaming experience around a customized instrument, which provides users with an augmented real environment representing information that would be otherwise invisible. In this specific case, the objects of observation are air pollutants emitted by everyday objects (e.g., sprays, candles, glue tubes), whose concentrations are measured in real time by a mobile sensor node.

**Figure 1:**
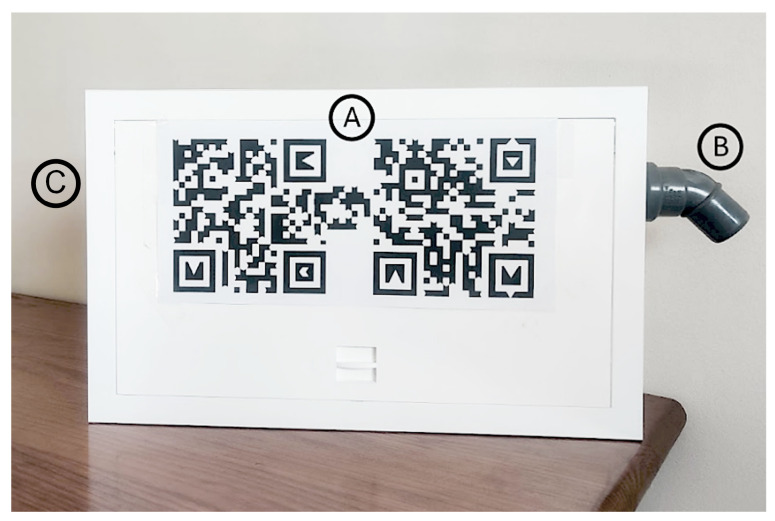
Air quality monitoring device. A—AR marker; B—air inlet tube; C—air outlet.

The game is based on a simplified version of the sensor node developed in the ExpoLIS project [8], which is able to analyze the concentrations of carbon monoxide (CO) and nitrogen dioxide (NO_2_), as well as of particulate matter with an aerodynamic diameter ≤1μm (PM1), ≤2.5μm (PM2.5), and ≤10μm (PM10). Airflow through the sensors is established by a fan attached to the air outlet inside the sensor node’s box. Figure 1 depicts the sensor node’s box used by the game. Its dimensions are as follows: 42cm wide, 26cm high and 9cm deep. The frontal face of the sensor node’s box includes an AR marker that allows the game to track it over time and augment it with virtual graphical representations.

**Figure 2:**
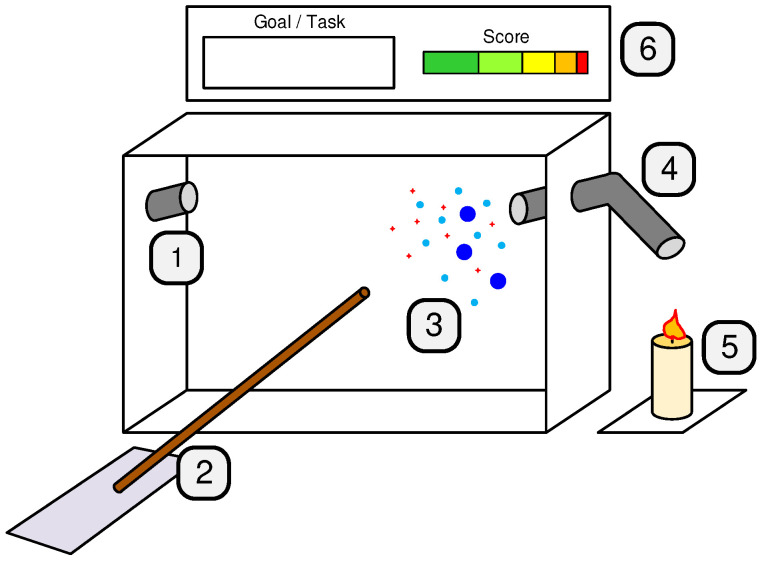
Gaming experience illustration. 1—air extraction tube (virtual); 2—“wand” for interaction with the experience (mixed); 3—representation of different air pollution compounds (virtual); 4—air inlet tube (real); 5—area for placing sources of pollution (real); 6—goals/score chart UI (virtual).

Figure 2 illustrates the gaming experience. The frontal face of the sensor node is rendered transparent using AR, allowing the user to see inside the box, which can represent, for instance, a room. Then, the user is asked to place an everyday object near the air inlet, e.g., a candle. The sensor node’s box analyzes the air and the detected pollutants are presented to the user as augmented graphical representations inside the box. Then, the user is asked to clean the air inside the box by using a set of plausible tools, representing actual air quality improvement techniques. These tools are selected and maneuvered with a virtual “wand”, which is controlled by the user pointing their hand.

### 3.2. Design Methodology

Due to the cross-disciplinary nature of serious games, their design and validation should involve people from the application domain (in our case, researchers on air quality), from the educational sciences (in our case, teachers), from computer science and engineering (for the actual development of the game), and, most importantly, representatives of the target user (in our case, children). Therefore, the development of this experience followed a participatory design with short prototyping and testing cycles. It resorts to testing small game components in small groups of users belonging to the target audience. This methodology allows one to collect users’ opinions and reactions to the tool, validating small development iterations in a practical and objective way. It avoids major setbacks in later stages of development since problems are identified earlier. It also stimulates new ideas and allows one to understand which features are most valued by the users. Therefore, user tests were carried out according to a task-oriented script during both the *formative* and *summative* evaluation phases [45], hereafter called *formative tests* and *summative tests*, respectively. During the tests, users were encouraged to *think aloud* [45], externalizing their thoughts during the experience.

The software components were developed using the widely known game engine Unity 3D [46]. Versatility and ease of use are two of its key features that contribute to its popularity. In addition, it is available for free, it has a vast community of active users on web forums, and it is well documented. Vuforia [47] was used for including the augmented reality components of the game. It is a C++ SDK dedicated to the creation of virtual environments interacting with the real world, and can be easily integrated with Unity 3D. Although Vuforia offers a wide variety of tools, only image recognition tools were used in the scope of the game. The goal is to recognize pre-defined markers (patterns) that, among other things, facilitate the sensor node’s box detection and its pose estimation. All 3D modeling was performed in Blender [48].

### 3.3. Pollution Compound Representation

As a result of the participatory design sessions, individualized representations of CO, NO_2_, PM1, PM2.5, and PM10 were considered excessive for the education level of the target audience (first cycle of basic education). Therefore, the design of the gamified experiment addresses the distinction between gases and particles, without emphasizing their individual classifications, easing the transmission and assimilation of knowledge by the target audience.

**Figure 3:**
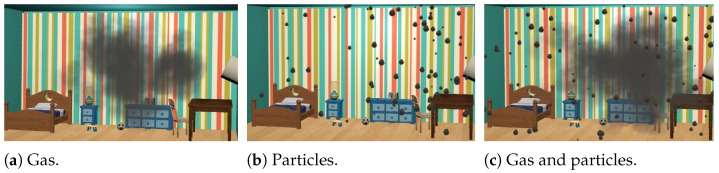
Graphical representation of gases and particles.

The unity particle system was used for creating representations of gases and particles. The particle system filled all the necessary requirements for this experiment since it incorporates physical components with several easily configurable properties: the number of emissions per second, emission velocity, reactions to the application of forces and collisions. The representation of gas and particles can be observed in Figure 3. Both are represented in gray, contrasting with the colored background, associating a negative connotation as if they were an enemy to be eliminated.

The emission value in units per second of each pollution element was assigned to one of four possible levels: 1 (low), 2 (moderate), 3 (intense), and 4 (extreme). The emission level assignment is directly related to the measurements retrieved by the sensors and is inspired by the *air quality index* (AQI), defined in [49]. AQI provides information about air quality, using a six-level scale that is easy to interpret. The representation of the pollutants only used the four highest levels from the AQI scale since sensor measurements always fell on those levels in the context of our experiments.

An initial displacement vector is assigned for both the representation of gases and particles. This vector is originated from the air inlet tube and points towards the opposite room wall. To simulate a realistic behavior of the compounds in the air, random displacement vectors are assigned during their lifetime to mimic the suspension of gas and particles in the air, moving slowly according to randomized flows.

### 3.4. User Interaction

#### 3.4.1. User Roles

As the experience was designed to be experienced by two children simultaneously, it is essential that both participants feel that they take an active part and play a well-defined role. Additionally, it was expected that the collaborative effort between the participants could enhance the outcome of the educational component of the game. Based on these premises, two user roles were defined: User1—selection and manipulation of real-world objects close to the sensor box air inlet; and User2—selection and manipulation of tools for interacting with the polluting compounds represented on the screen.

The interaction associated with the role of User1 is to use a set of real-world objects in order to cause a reaction from the sensor box. The available set of objects include: deodorant spray, a dusting cloth, a candle, and a tube of liquid glue. Each object has its own way of being operated in order to spread polluting compounds into the air: the cloth is shaken, the candle is lit, and so on. With the exception of the cloth shaking, which is used as an example in the starting tutorial, the discovery of the actions that cause reactions from the sensor box is left to the users, helping them only if necessary. When correctly operating an object close to the box’s air inlet, the representation of the pollution elements released by the object (gas, particles, or both) show up on the screen. The role of User1 remains the same throughout the experience: the user is responsible for selecting the objects to use at each moment, producing the polluting compounds necessary to achieve the objectives presented during the game run. Since the use of cloth only guarantees the introduction of particles into the environment, the in-game objectives and scoring system will induce the need to further explore the remaining objects in order to discover how to use them and which ones produce gases.

The actions of User2 begin as soon as the first polluting elements are presented in the screen. This user’s role is to clear the air, removing the pollution compounds using a set of tools at one’s disposal. These tools are used for directing the gases and particles to the sensor node’s air outlet, represented by a virtual window in the augmented sensor node. To learn how to relate and operate each tool with each type of pollutant, the user needs to carry out some experiments. The iterative design of User2’s interactions, informed by interleaved formative evaluations, is described in the following paragraphs.

#### 3.4.2. Early Design

During the initial development phase, two kinds of pointing mechanisms were considered: mouse vs. the user’s hand. The objective was to test the response of the target audience to this interaction type whilst consuming the least amount of resources and time as possible. The interaction with the virtual environment was achieved through collisions of the element controlled by the pointing device with the game elements, enabling the possibility of selections (e.g., pressing buttons) and object manipulation (e.g., dragging).

The use of a mouse as an interaction device was considered due to its wide spread use and, thus, potential smooth learning curve. The 2D mouse movement was mapped to a 2D movement in a vertical plane aligned with the box, meaning that it did not allow the user to control the depth. Mouse rotation was not considered.

**Figure 4:**
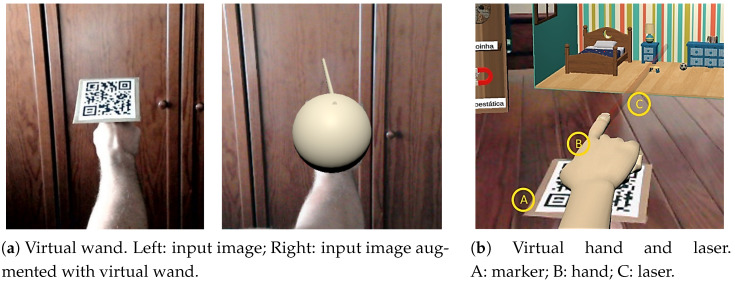
Pointing mechanism: early design in (**a**) and final design in (**b**).

Pointing with one’s hand is natural and, thus, potentially more intuitive than using the mouse. As in previous work [50], we track the user’s hand using AR makers. Concretely, to track the user’s hand when pointing to the sensor node, a rigid AR marker was glued to a ring that could be used in the index finger. The marker was used to track the pose of the finger (position and rotation), interact with other virtual objects according to the laws of physics, and overlay a virtual object representing a *virtual wand* (see Figure 4a).

**Figure 5:**
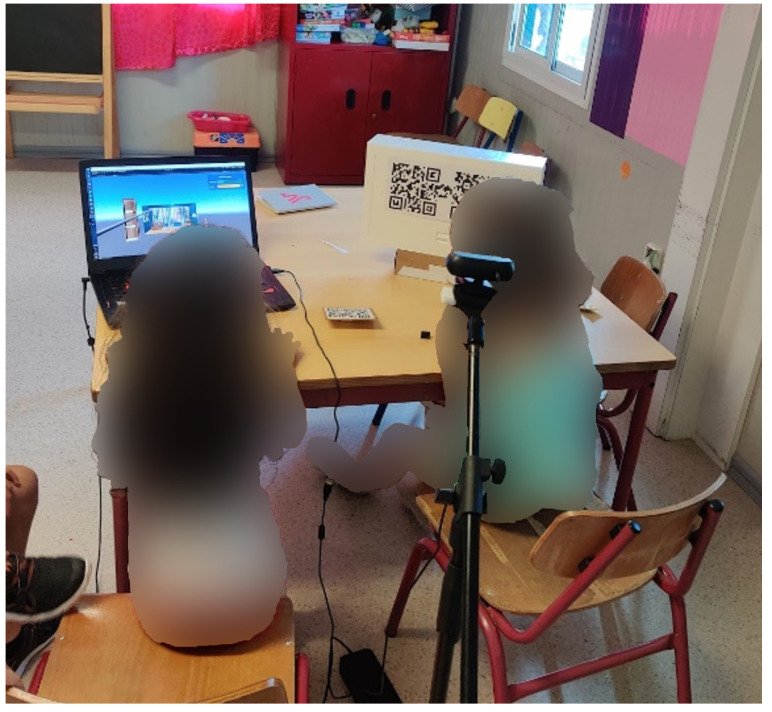
Two children playing during the formative evaluation.

Formative evaluation with nine children highlighted a set of flaws in the early design of the marker-based virtual wand (see Figure 5). All participants presented difficulties in perceiving the depth of the virtual objects in relation to the virtual wand. In some cases, participants were not even sure that they were pointing to the sensor node altogether. On the other hand, occasional failures in tracking the markers (box and ring) was well accepted by all participants, which interpreted these events as part of the game challenges. After some trial-and-error, participants devised strategies to avoid failures and facilitate the re-detection of the markers. Behaviors such as bringing the marker closer to the camera and keeping it parallel to the image plane were increasingly frequent.

Six of the nine users reported having a preference for interaction using the mouse over the method with the marker. The children successfully completed the assigned tasks much faster when using the mouse. The learning curve of using the ring with the marker showed to be longer than using the mouse. On the other hand, the experience became less interactive and challenging when using the mouse.

The users’ astonishment reaction was evident when the ring method was presented to them; however, this sensation was soon lost due to the difficulty in handling it. By analyzing the results of the formative tests, the poor results for using the marker’s ring interaction were not justified by a limitation of the method itself, but by the characteristics of its implementation. The following paragraphs describe the set of improvements that were implemented in order to cope with the found limitations.

#### 3.4.3. Final Design

To provide better control over the pointing direction, the context was improved by superposing a virtual hand with a pointing finger on the marker and the virtual wand was substituted by a virtual laser beam. The laser beam provides a continuous visual direction cue from the hand to the screen, facilitating the user perception by requiring less saccades. Figure 4b depicts the final design.

The children’s difficulty for dealing with depth during the interaction was tackled by reviewing the mechanics of the experience. In short, interactions along the depth axis were simplified and restricted to a thin slice of the box in the virtual 3D world. Thus, depth variations associated with the movement of gases and particles were set to a much smaller range, making the interactions with those elements closer to a two-dimensional case. Gases and particles bounce back when the boundaries of the box slice are reached.

The manipulation of pollution removal tools along the depth axis was also constrained in order to ease the intersection with the virtual pollutants. For this, an invisible ray is cast along the user’s pointing direction and its intersection with the longitudinal plane that splits the slice (defined in the previous paragraph) in half is considered. The tool is then positioned at the intersection point, ensuring that its movement is always performed along the slice splitting plane. Overall, users showed less confusion interacting with the system after these changes took place, and thus, these were included in the final prototype.

### 3.5. Air Pollution Removal Tools

**Figure 6:**
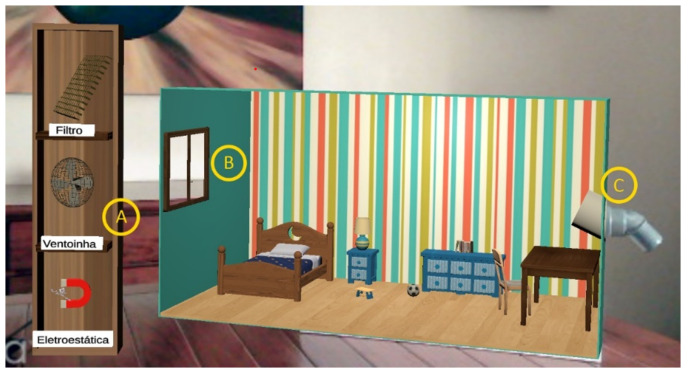
Virtual toolbox and sensor box. A—toolbox; B—particle and gas extraction window; and C—sensor air inlet.

To guarantee the pertinence and integrity of the information transmitted by the gamified experience, the visual representations of the air pollution removal tools and their interactions with polluting compounds were analyzed in the participatory design sessions. During gameplay, these tools are available to the user on the left side of the sensor node, as illustrated in Figure 6. The importance of aeration in good air quality is taken into account by including a virtual window next to the sensor node’s air outlet. Users are expected to remove pollutants through this window.

To select a tool, the user only needs to point to its direction. A tool is replaced if another is selected. Explanatory text associated with the tools was not included, leaving it up to the user to try them out and autonomously discover their features. When a tool is selected, it is coupled to the end point of the virtual laser beam representing the user’s pointing direction. The position and orientation of the selected tool is then controlled by the user according to the pose of the finger-mounted marker. Only slight adjustments are applied to the rotation angles relative to the marker in order to make the interaction more natural. As such, the user is able to manipulate the tool to direct the polluting compounds to the extraction point (i.e., the window).

Table 1 summarizes the implemented set of tools (filter, fan, and electrostatic) as well as the list of pollution compounds with which each of them is able to interact. The diversity of tools and air pollutants was judiciously selected in order to ensure that, while playing the game, children learned about air pollutants and their countermeasures.

**Figure 7:**
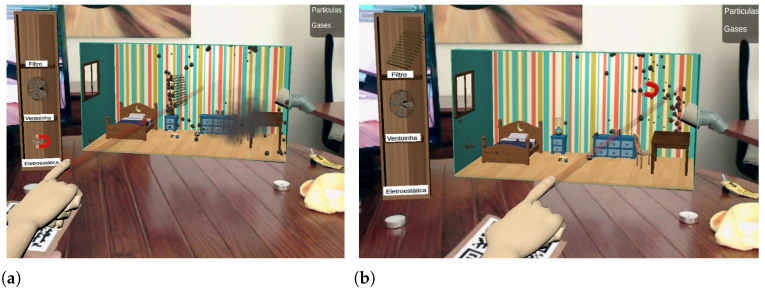
Interaction with the filter tool (**a**) and with the electrostatic tool (**b**).

With the *filter tool*, the user is able to collect coarser particles, having no effect on gases and finer particles. This binary behavior is a simplification of real-filtering devices, which use meshes to retain particles as a function of their diameter. Figure 7a shows this tool in action.

**Figure 8:**
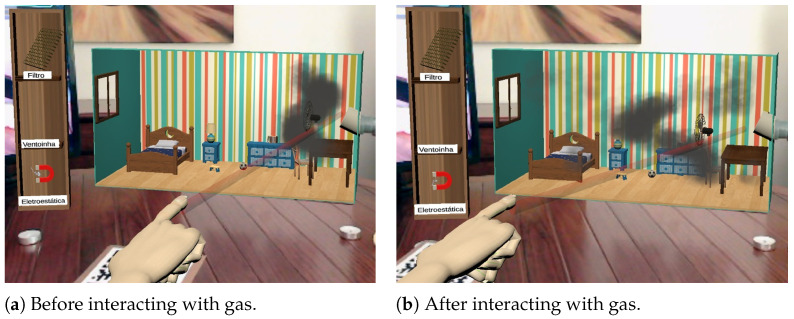
Interaction using the fan tool.

The *fan tool* creates an air flow along the direction it is pointed in, influencing the movement of all polluting compounds. The tool is implemented as a repulsive force field, of limited range, that interacts with the particle systems controlling the virtual air pollutants so as to push them away along the fan direction. This tool, represented in Figure 8, has a greater effect on gases and smaller particles, as these have a lower mass value.

The *electrostatic tool* represents a device, known as electrostatic precipitator, that induces electric charge into the particles, capturing them by electromagnetic attraction. This tool was implemented as an attractive force field, of limited range, influencing the virtual pollutant particles so as to pull them towards the tool. The representation chosen for this technique is the least faithful among the other tools. Instead of considering a graphical representation of an electrostatic precipitator, which is a device not familiar to children, its representation ended up in the form of a magnet. When used, it emits small “sparks” (Figure 7b) to highlight the presence of electrical charges, in order to induce the users to establish a relation between its representation and its operating principles.

### 3.6. Spatial Setup

**Figure 9:**
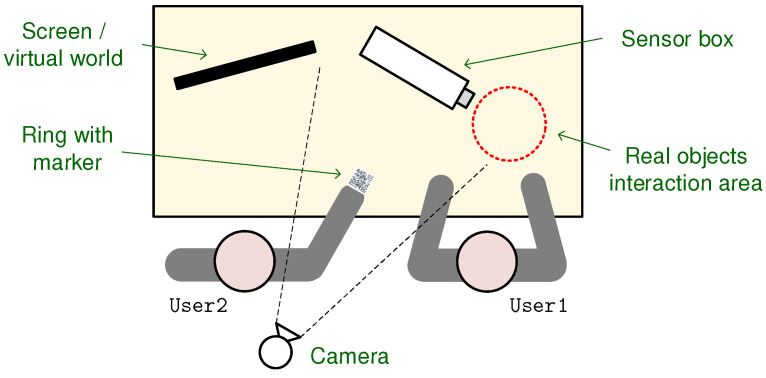
Spatial layout.

The setup is composed of a sensor node, a desktop computer, a computer screen, and a USB camera, spatially arranged as depicted in Figure 9. In this layout, the screen and the box are placed side by side, with the camera at the opposite end, facing the box. Among several alternatives tested during the formative evaluation, this spatial arrangement shows to be the most adequate. This decision took into account: (1) position and orientation of the camera so as to capture real-world content and to define the viewing perspective over the virtual elements; (2) position and orientation of the screen where real and virtual world components are combined; and (3) user placement and their interactions with the system.

Both camera and screen were placed at fixed locations. This arrangement frees the children’s hands for interaction with real objects and markers. In addition, by keeping the camera still, we avoid a set of technical challenges that could impact upon the user experience, such as distractions emerging from dynamic backgrounds, marker tracking issues resulting from variations in the illumination conditions, and jitter resulting from the varying frame rate due to the increasing complexity of handling dynamic settings.

Given that the screen and the sensor node were placed side-by-side, the former was presented as a “magic mirror”, through which it was possible to see things inside the box that are not visible to our eyes. However, mirroring the captured image, for the screen to indeed be a mirror, proved to be an added difficulty for interacting with the marker. For this reason, the mirror metaphor was tested without actually mirroring the image, to which users responded positively. The solution was well accepted, resulting on easier and more intuitive interactions when compared with other tested setup possibilities.

### 3.7. Game Mechanics

#### 3.7.1. Story Line

**Figure 10:**
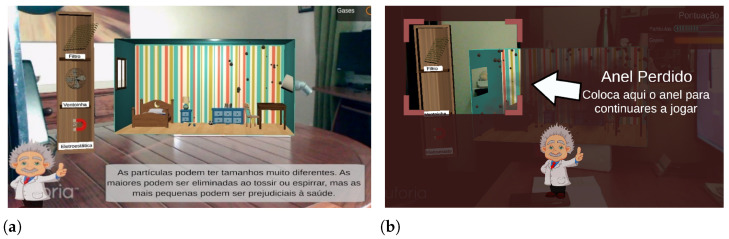
Interaction with the NPC. (**a**) A screenshot of the game with the scientist NPC and its subtitles (in Portuguese) at the bottom. Subtitles translation: *Particles can have very different sizes. The larger ones can be expelled by coughing or breathing, but the smaller ones can be harmful to our health*. (**b**) Assistance screen shown when the ring marker is lost. Overlaid text translation: *Ring lost. Place the ring here to continue playing*.

To guide the experience and reinforce its didactic content, a non-playable character (NPC) was created. The NPC was a scientist, graphically represented with sprites, who appears only in key moments of the experience. The NPC communicates (in Portuguese) with the user via subtitles, to provide hints on how to interact with the experience and to provide additional data that may help the user understand what is being observed. Figure 10a shows one of these situations, in which the NPC appears with pedagogic information regarding the particles that have just appeared.

The NPC also intervenes in a small tutorial presented at the beginning of the gaming experience and when problems are detected, namely when the ring marker is not detected for over five seconds. In the latter case, the screen shown in Figure 10b is displayed, telling the user what action must be done to resume marker tracking and, thus, playing. This screen displays an area where the user must place the ring within a strategically chosen position that avoids occluding the sensor node’s marker. This display is accompanied by an audio feedback with negative intonation.

#### 3.7.2. Scores and Feedback

**Figure 11:**
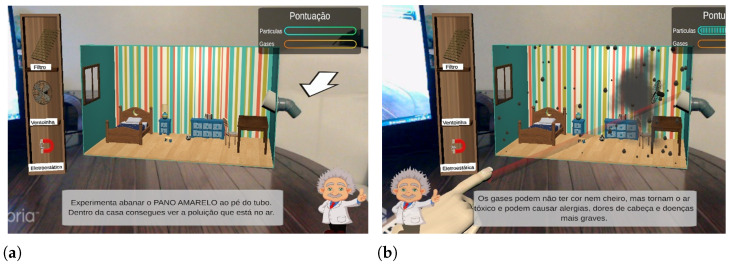
Interaction with NPC. (**a**) NPC giving the initial tutorial. Subtitles translation: *Try shaking the yellow cloth near the tube. Inside the house you can see the pollution that is in the air*. (**b**) NPC providing information after the first release of gas/particles. Subtitles translation: *Gases may be colorless and odorless, but they are toxic and can cause allergies, headaches and more serious illnesses*.

The gamified experience comprises a scoring system, which rewards the user whenever gases or particles are directed to the window. This intends to be a simple way of providing feedback and assigning tasks to users, encouraging them to use different objects and tools in order to collect points. The score information is displayed in the upper right corner of the screen (see Figure 11a), and the increment in each score bar is accompanied by audio feedback with positive intonation.

Dynamic difficulty adjustment (DDA) is used to ensure the inclusion of users with less experience or showing greater difficulties adapting to the system, reducing the possibility of frustration. Additionally, DDA helps keep the duration of the experience between 7 and 10 min, which is the empirically found duration for both users to explore the game elements and feel comfortable with the game mechanics to successfully complete the objectives.

Each time the user manages to expel a particle through the virtual window, they earn 2% of the maximum possible score. Conversely, by expelling a gas unit, which is easier than expelling particles, the user earns 0.5% of the maximum possible score. If the user presents significant difficulties in scoring or achieving the objectives assigned during the experience, DDA takes place by: (1) doubling the future particle emission levels; and (2) doubling or even tripling the future earned score increments. Boosting particle emission levels raises the chances of successful interaction with particles, whereas boosting score increments reinforces positive feedback.

#### 3.7.3. Gameplay

Once they arrive at the game location, users choose the role they will play in the experience without realizing it through the chair they sit on. In the first contact with the experience, a brief contextualization is made. It is explained that the screen shows the image captured by the camera (which is on their back) and the way in which the markers work, highlighting the connection between the real world and the virtual world by moving the sensor node’s box (the term ’box’ was used for the sake of simplicity), making it noticeable that it moves similarly in the virtual world. It is then explained that a sensor was placed inside the box that identifies small compounds in the air that we cannot see. If the users have no questions, the experiment starts.

At the beginning of the experiment, the screen is presented as a magic mirror, where it is possible to observe the otherwise invisible polluting compounds. This is followed by the mini tutorial given by the NPC, which suggests to the user that they shake the cloth close to the box’s air intake tube (indicated in the screen with an arrow), as can be observed in Figure 11a. As soon as the user does so, the first particles appear in the virtual environment, about which the NPC makes a small theoretical introduction. The interventions of the NPC when the first particles and gases are introduced can be observed in Figure 11b. At this point, the child assuming the role of User2 has already performed some experiments and realized how one can interact with the virtual elements by pointing at them. The selection of pollution removal tools was typically tried out, as motivated by children’s intrinsic curiosity. Even if User2 does not demonstrate the desired autonomy for selecting a tool and using it to interact with the polluting compounds, some of the compounds eventually end up reaching the window and triggering a positive feedback sound effect. From this moment on, it is expected that both users will explore the tools/objects at their disposal, in order to fill in the score bars completely. Although exploratory freedom is given to the users, these are not allowed to place more than one object simultaneously in the proximity of the sensor’s air intake, a situation that was frequently observed during the formative evaluation.

## 4. Evaluation

### 4.1. Method

In the validation phase of the tool, based on summative tests, 27 children aged between 7 and 11 years old, belonging to two primary schools in Sintra, Portugal, participated with parental consent. We sought to obtain a group of participants with a uniform distribution of ages (μ=7.75, σ=0.93) and genders. Although the experiment was carried out in pairs and the questionnaires were answered simultaneously, the participants were sufficiently distanced not to hear each other’s answers. An adult read the questions and provided explanations when needed.

To assess satisfaction and what knowledge had been gained about air pollution as a result of playing the game, three questionnaires were answered by the participants: a pre- and post-game questionnaire to assess knowledge about air pollution; a post-game satisfaction and usability questionnaire; and a post-game open-ended questionnaire about participants’ opinions and preferences.

To assess the added value of including physical objects in the game, a variant of the game was tested. In this variant, instead of manipulating four physical objects, the user manipulated four markers, i.e., small images printed on paper, each representing one of the physical objects. These markers are automatically detected by the game whenever they fall inside the camera’s field of view. All participants experienced both versions of the game in a randomized order, as well as both forms of interaction with each version.

After answering the pre-game questionnaire to assess knowledge about air pollution, a brief explanation about the experience was given. This included using the physical objects and markers, as well as the notion that the box contains air pollution sensors inside, whose observations are graphically represented in the game’s screen.

Real-time communication between the game and the sensor node being assured, the validation of the pedagogical component of the game takes priority. To attain this goal, the mechanics of representation and interaction were isolated from the physical box elements. The uncertainty of the readings and the need for calibration are examples of factors that need to be accounted for in the experiments. To ensure the predictability of the system and, consequently, reduce the variance in the experimental data, the Wizard of Oz method [45] was used. This method consists of simulating the reactions of the system under study using covert human actions, leading the user to believe that the reactions are being produced by the system. In our case, the presence of air pollutants emitted by the objects placed by the user near the air inlet tube is covertly reported to the game by a human and not by the actual sensors of the physical box. To apply this method, a wireless keyboard was used and a set of virtual keys were mapped in order to simulate different levels of pollution emissions from each of the elements. The keyboard is used by the person conducting the test, imperceptibly to the user. This allowed to avoid configuration and calibration processes, saving time for the game development, without jeopardizing the gamified experience validation.

#### 4.1.1. Knowledge Validation Questionnaire

The Knowledge Validation Questionnaire, validated by a teacher of basic education, consists of four questions that are answered by the participant before and after playing the game. The goal of this questionnaire is to assess whether playing the game results in knowledge gain regarding air pollution. To reduce stress among the participants, the questions were orally presented to the children, instead of using the written format. The children’s oral responses were audio-recorded for offline analysis.

The questionnaire’s four questions (translated from Portuguese to English) are as follows:

- Q1: *Do you think there could be air pollution inside our homes?* Response: Yes/No.
- Q2: *What do you think can pollute the air inside our homes?* Response: An enumeration of objects.
- Q3: *What do you think is in the air when it’s polluted?* Response: An enumeration of air pollutants.
- Q4: *How do you think we can clean up the air pollution we have inside our homes?* Response: An enumeration of cleaning methods.

Questions Q2–Q4 were only asked if the participant responded “yes” to Q1.

#### 4.1.2. Satisfaction and Usability Questionnaire

The Satisfaction and Usability Questionnaire (SUQ) was answered by the participants after playing the game with the goal of assessing: (1) their intention to repeat the gaming experience (repetition is important to help consolidate knowledge); (2) their perception regarding how easy/intuitive it is to interact with the game (a non-intuitive experience can hamper learning); and (3) their perception of the game’s utility as a learning tool.

SUQ consists of eight statements adapted from the standard questionnaires SUS [51] and TAM [52], which participants were asked to classify using a five-point Likert scale, ranging from “1 (I totally disagree)” to “5 (I totally agree)”. A visual representation of the Likert scale was used, as suggested in an adaptation of the SUS for children [53]. The statements composing this questionnaire (translated from Portuguese to English), as well as their goals for the analysis, are:

- S1: *If we had more time, I’d like to keep playing the game*. Goal: assess users’ immersion in the game, levels of acceptance, enjoyment, and whether it captivates exploration.
- S2: *I would like my teacher to use these types of games in the classroom*. Goal: assess the participant’s perception of the game’ utility as a teaching tool compared to traditional teaching methods.
- S3: *If I had this game at home I would like to play it a lot more*. Goal: assess the participant’s interest in the game outside the classroom context.
- S4: *I felt confused several times while playing*. Goal: assess the participant’s perception of ease of use and interaction, as well as of harmony between the real and virtual world.
- S5: *To play this game I feel like I need an adult’s help*. Goal: assess the participant’s sense of autonomy during the gaming experience, the learning curve, and the interaction complexity.
- S6: *If I played this game more often, I would learn a lot more about pollution*. Goal: assess the participant’s perception of knowledge acquired as a result of playing the game.
- S7: *My friends will really like this game*. Goal: call for a deeper analysis of the levels of satisfaction and enjoyment, asking the user how the game will be perceived by a more general group.
- S8: *My friends will learn a lot about pollution with this game*. Goal: call for a deeper analysis of the game’s ability to transmit knowledge, asking the user how the game will be perceived by a more general group.

To avoid biases, statements in the original SUS are posed with both positive and negative formulations. However, the successive change in the connotation of the questions can create some confusion in the user. In fact, during a set of formative tests, we found that this factor is even more evident in children, as they often provided random or nonsensical answers when they did not fully understand the questions. To avoid these issues, our statements are all positively formulated. This is supported by existing evidence that questionnaires can be equally valid when only statements with positive formulations are used [54].

#### 4.1.3. Questionnaire of Opinion and Preference

After answering the SUQ, participants were asked to answer to a final Questionnaire of Opinion and Preference (QOP). This questionnaire is composed of four open questions whose purpose is to validate the data collected with the previous questionnaires, elicit additional information, and assess which components of the gaming experience are most appreciated. The questions composing this questionnaire (translated from Portuguese to English), as well as their goals for the analysis, are:

- Q1: *Do you prefer to play the game with real objects or with cards? Why?* Goal: assess the participant’s perception of the importance of including physical objects and sensors in the gaming experience.
- Q2: *Do you think the game is useful for learning? Why?* Goal: assess the participant’s perception of knowledge acquired as a result of playing the game.
- Q3: *Would you like to learn other subjects with this type of game? Which ones?* Goal: assess the participant’s perception of the utility of AR-based games in learning contexts.
- Q4: *What did you like most and least about the game?* Goal: pinpoint the elements that deserve further attention when developing AR-based learning games.

### 4.2. Results

#### 4.2.1. Knowledge Validation Questionnaire

As a result of questions Q2–Q4 of the Knowledge Validation Questionnaire only being asked to participants who answered yes to Q1, those three questions were answered by only 10 and 21 participants in the pre- and post-game questionnaires, respectively.

The answers to *Do you think there could be air pollution inside our homes?* clearly show that the game allowed participants to learn about air pollution. Concretely, Table 2 shows that the proportion of participants acknowledging that air pollution may exist inside their houses is significantly higher (p<0.001, n=27) after playing the game (77.8%) than before playing the game (37.0%). This knowledge gain highlights the importance of learning tools as the one presented. Importantly, none of the participants who answered affirmatively in the pre-game questionnaire changed their opinion in the post-game questionnaire, which seems to indicate that the game did not induce confusion.

The answers to the question *What do you think can pollute the air inside our homes?* were mostly categorized as related to the emission of gases or to particulate matter. For instance, terms like *smoke* and *fireplace* were (mostly) categorized as (related to) *gases*, whereas terms such as *cloth* and *sweep* were categorized as *particles*. The goal was to determine the extent to which participants were aware that the air quality is influenced by objects and people’s behaviors that do not necessarily result in the production of gases. Table 3 shows that all participants were unaware of this relationship before playing the game. Remarkably, after playing the game, 22.2% of the participants provided answers using expressions categorized as particles. In the same line, the percentage of participants mentioning expressions related to gases roughly doubled after playing the game. The table also shows that the percentage of participants that did not know how to respond, provided invalid responses, or failed to recognize that there is pollution inside our homes, was roughly halved after playing the game.

The answers to the question *What do you think is in the air when it’s polluted?*, summarized in Table 4, are aligned with the answers provided to the previous question and revealed a considerable knowledge gain after playing the game. The percentage of participants that did not know how to respond, provided invalid responses, or failed to recognize that there is pollution inside our homes, reduced by approximately two thirds after playing the game. Additionally, the percentage of participants that provided answers using expressions categorized as particles and gases was roughly six and five times higher after playing the game, respectively. Hence, Table 3 and Table 4 show that the game contributed to the enhancement of participants’ knowledge and self-confidence.

The percentage of participants providing valid answers to question *How do you think we can clean up the air pollution we have inside our homes?* increased from 22.2% before playing the game to 55.6%, after playing the game. The results show that the children were able to improve their knowledge on this topic. Concretely, the references to *ventilation* and related words used by the children raised from 7.4% (pre-game) to 22.2% (post-game). This is aligned with references to the explicit use of a *fan*, which increased from 0% (pre-game) to 18.5% (post-game). Unfortunately, the other tools were explicitly mentioned by only one of the participants. This means that the fan is notoriously the most memorable tool in the game.

#### 4.2.2. Satisfaction and Usability Questionnaire

Table 5 presents the 95% confidence intervals associated with SUQ responses. The closer to five the score in each statement of the questionnaire is, the better the game has been experienced by the participants, except for two statements (marked with * in the table), for which a score closer to one is better. All obtained scores are between the ideal scale extreme and the scale’s mid-point, meaning that the game is globally perceived as satisfying, usable, and educationally enriching. This conclusion is reinforced by the positive results obtained around the three aggregating factors presented in Table 6: (1) intention to use again; (2) perceived learning utility; and (3) perceived easiness of use. Interestingly, it was observed that all pairs of participants explored all objects and all tools, even when only some of them were required to complete the given game goals.

#### 4.2.3. Questionnaire of Opinion and Preference

To the question *Do you prefer to play the game with real objects or with cards?*, the majority of participants (85.2%) stated to prefer the version of the experience with real objects over the version in which these objects were replaced by paper card representatives (see Table 7). This result confirms the value of the tangible interface of the game based on the interaction with physical objects. When asked why they prefer physical objects over paper cards, participants revealed four main reasons summarized in Table 8: more realistic (33.3%), better control (29.6%), easier perception, (14.8%), more fun (11.1%), while 11.1% were unable to provide a reason. Conversely, the participants who stated to prefer to interact with paper cards over physical objects (14.8%) justified this preference due to the easiness of interaction.

To the question *Do you think the game is useful for learning?*, all participants answered affirmatively. These results are in agreement with the results obtained in the SUQ. The same trend has been observed in the answers to the question *Would you like to learn other subjects with this type of game?*, to which all participants but one answered affirmatively. Hence, we can conclude that participants perceive playing AR-based learning games to be an enriching learning activity whose application spectrum is not limited to air quality. The topics that participants reported to be ones they would like to see addressed by AR-based learning games were mostly related to pollution and waste processing, but also animals and forest. Other less frequently reported preferences include the human body, the universe, mathematics, dinosaurs, and cooking.

Participants were unable to provide a significant amount of answers to the question *What did you like most and least about the game?*. This may mean that the question is too open for the participants’ age group or that they were already tired in the end of the testing session. Nevertheless, we believed it was worth analyzing the provided responses.

The most frequent responses regarding the most liked aspects of the game included the terms *fan*, *candle*, *ring*, *smoke*, and *winning*. These highlight the interest in the augmented reality (*ring*), the gaming component (*winning*), the toy-like characteristics of the game (*fan* used for clearing *smoke*), and the importance of using interesting real-world objects (*candle*).

The least liked component of the game was the *filter*, possibly because it was the less familiar item. *Interaction with cards* was also mentioned by participants, since they strongly preferred playing using real-world objects. This is a positive result that supports the importance of considering real objects as tangible interface. *Little time to play* was also among least liked aspects. This was also an interesting response, as it suggests that participants were willing to play for longer periods.

### 4.3. Discussion

The results show that playing the AR game results in knowledge gain regarding the causes of indoor air pollution (objects and people behaviors) and about its mitigation mechanisms, thus confirming research hypotheses H1 and H2. This knowledge gain is particularly evident regarding the influence of particulate matter on air quality (in comparison to gases) and on the importance of house ventilation to improve air quality.

The results also confirm research hypothesis H3 by showing that the AR game is perceived by children as useful for learning, easy to use, and providing a satisfying experience that they are willing to repeat. These results are important because learning to be effective requires repetition and a game with low replay value is not able to deliver this.

All participants explored all objects and tools, even though they were not required to complete the game. We believe that this desirable exploratory behavior is at least in part due to the physics-based mechanics of the game, which was included to leverage on the children’s intrinsic motivation towards toys. If provided with toy-like properties, a game should become more interesting to play with, even before the player knows exactly what game’s goals are [7].

Finally, the results show that participants prefer using real objects as a tangible interface rather than card-based representatives, thus confirming the research hypothesis H4. The realism and easiness of interaction were the most indicated reasons. This shows the value of augmented reality, enhancing the physical reality with digital content, rather than substituting it altogether.

## 5. Conclusions

A serious game for teaching children about air pollution was presented. More than simply conveying the dangers of air pollution, the game aims to inform children about the several types of air pollutants, what causes them, and which mechanisms are available to clear polluted air. To accommodate these goals, the children are invited to interact with a physical sensor node in order to playfully correlate everyday objects and the air pollution emitted by them. This tangible form of interaction aims to render the learning of messages more memorable and plausible. The game exploits augmented reality to provide children with the possibility to effortlessly view the invisible, that is, the air pollutants emitted by the everyday objects manipulated by them, further reinforcing the learned messages. To boost learning and enjoyment via socialization, children play the game in pairs.

A participatory design process was followed with intermediate formative evaluation moments to ensure that the design meets the desirable goals. The final design was subject summative evaluation with a sample of 27 children aged between 7 and 11 years using the Wizard of Oz method. The results show that playing the game is an effective learning experience. Concretely, comparing pre- and post-game data, the results show that playing the game allowed most children to better recognize that air pollution may exist inside our homes, air pollution is not only composed of gases but also of particulate matter, garbage and air pollution are not the same, and that ventilating our homes is important. The results also show that most children felt that the game was useful, usable, and enjoyable, resulting in an intention play again. In fact, all participants stated that they would like to use this type of game to learn other subjects. Finally, the results also show that most children preferred playing with real everyday life objects than with card surrogates, mostly due to realism and control.

The game was tested with a sample size of 27, which is close to the usually considered threshold for the central limit theorem to hold (n≥30) [55]. Nevertheless, given that it is better to have a larger and more diverse sample size, we plan to expand our user study. As future work, we also intend to assess the potential long-term learning benefits provided by the game. For this, it will be necessary to design an experimental protocol in order to test the children’s knowledge after a prolonged period from the moment they played the game. We would also like to repeat the experiments without using the Wizard of Oz method, that is, using real data automatically collected by the physical sensor node. We also intend to expand the game in order to teach children about the impact of other human activities and behaviors on indoor air quality (e.g., cooking, house cleaning, incense burning, and fireplace use) and outdoor air quality (e.g., people and goods transportation, industrial activity). Raising children’s awareness of this impact is important because these activities emit air pollutants and are common in households and cities. From a technical standpoint, we plan to study the use of hand tracking techniques that do not require the use of a ring marker. We also consider it important to explore the use of visualization devices that would allow the game to be played on the move (e.g., AR glasses), beyond the tabletop, enabling the inclusion of location-based mechanics. Finally, we would like to expand the game with additional narratives, capable of attracting children of different age groups. Ideally, these narratives are inclusive and personalized to the children.

## Figures and Tables

**Table 1:** Tools for pollution compound removal.

Tools	Pollution Compound		
	Gases	<PM2.5	≥PM2.5
Filter	×	×	√
Fan	√	√	√
Electrostatic	×	√	√

**Table 2:** Response distribution (yes/no) to question “Do you think there may be air pollution inside our homes?”

	Yes	No
Pre-game	37.0%	63.0%
Post-game	77.8%	22.2%

**Table 3:** Percentage of the 27 participants whose answers to the question “What do you think can pollute the air inside our homes?” mostly included expressions related to either particles (first column) or gases (second column). Some participants included expressions related to both types of air pollutants in their responses. The third column presents the percentage of participants which reported that they did not know how to respond (D), provided invalid responses (I), or failed to recognize that there is pollution inside our homes (U).

	Particles	Gases	D + I + U
Pre-game	0.0%	29.6%	70.4%
Post-game	22.2%	63.0%	37.0%

**Table 4:** Percentage of the 27 participants whose answers to the question “What do you think is in the air when it’s polluted?” mostly included expressions related to either particles (first column) or gases (second column). Some participants included expressions related to both types of air pollutants in their responses. The third column presented the percentage of participants that reported they did not know how to respond, provided invalid responses, or that failed to recognize that there is pollution inside our homes.

	Particles	Gases	D + I + U
Pre-game	3.7%	11.1%	85.2%
Post-game	22.2%	55.6%	29.6%

**Table 5:** 95% Confidence intervals (CI) for the results obtained with the Satisfaction and Usability Questionnaire, answered with a five-point Likert scale (from 1 to 5). Note that, in opposition to all other statements, the lower the values obtained for S4 and S5, the better. These are annotated with *.

Statement (Summarized)		95% CI
S1	*I’d like to keep playing this game*	[4.61, 4.95]
S2	*I’d like my teacher to use these types of games*	[4.17, 4.94]
S3	*I’d like to play this game again at home*	[4.08, 4.66]
S4	*I felt confused while playing this game*	* [1.70, 2.60]
S5	*I feel I need adult’s help to play this game*	* [1.36, 1.90]
S6	*I’d learn more if I play this game more*	[4.19, 4.70]
S7	*My friends will like to play this game*	[4.42, 4.91]
S8	*My friends will learn with this game*	[4.10, 4.64]

**Table 6:** Results of the Satisfaction and Usability Questionnaire presented in Table 5 averaged (AVG) by factors. Note that, in opposition to all other factors, the lower the value obtained for *perceived easiness of use*, the better. This inverted value is annotated with *.

Factor	Statements	AVG
Intention to use again	1, 3, 7	4.61
Perceived learning utility	2, 6, 8	4.46
Perceived easiness of use	4, 5	* 1.88

**Table 7:** Distribution of the responses to the question “Do you prefer to play the game with real objects or with cards?”.

Paper Cards	Real Objects
14.8%	85.2%

**Table 8:** Distribution of the reasons presented by the participants to the question “Why did you prefer to use real objects over paper cards?”

Realism	Control	Perception	Fun	Do Not Know
33.3%	29.6%	14.8%	11.1%	11.1%

## Data Availability

The datasets used and/or analyzed during the current study are available from the authors on reasonable request.

## Abbreviations

The following abbreviations are used in this manuscript:

AQI	Air Quality Index
AR	Augmented Reality
CI	Confidence Interval
CO	Carbon Monoxide
DDA	Dynamic Difficulty Adjustment
HMD	Head Mounted Display
IoT	Internet of Things
QOP	Questionnaire of Opinion and Preference
NO_2_	Nitrogen Dioxide
NPC	Non-Playable Character
PM1	Particulate Matter with an Aerodynamic Diameter ≤1μm
PM2.5	Particulate Matter with an Aerodynamic Diameter ≤2.5μm
PM10	Particulate Matter with an Aerodynamic Diameter ≤10μm
SUQ	Satisfaction and Usability Questionnaire
SUS	System Usability Scale
TAM	Technology Acceptance Model
VR	Virtual Reality
WHO	World Health Organization
